# Lipid profile of bovine grade-1 blastocysts produced either in vivo or in vitro before and after slow freezing process

**DOI:** 10.1038/s41598-021-90870-8

**Published:** 2021-06-02

**Authors:** Sarah Janati Idrissi, Daniel Le Bourhis, Antoine Lefevre, Patrick Emond, Laurene Le Berre, Olivier Desnoës, Thierry Joly, Samuel Buff, Virginie Maillard, Laurent Schibler, Pascal Salvetti, Sebastien Elis

**Affiliations:** 1 Allice, 37380 Nouzilly, France; 2 UMR 1253, iBrain, Université de Tours, 37004 InsermTours, France; 3 CHRU Tours, 37004 Tours, France; 4 Université de Lyon, ISARA-Lyon, UPSP ICE 2016.A104, 69007 Lyon, France; 5 Université de Lyon, VetAgro Sup, UPSP ICE 2016.A104, 69280 Marcy l’Etoile, France; 6 CNRS, IFCE, INRAE, Université de Tours, PRC, 37380 Nouzilly, France

**Keywords:** Developmental biology, Embryology, Animal biotechnology

## Abstract

Currently, in vitro embryo production (IVP) is successfully commercially applied in cattle. However, the high sensitivity of embryos to cryopreservation in comparison to in vivo (IVD) embryos slows the dissemination of this biotechnology. Reduced cryotolerance is frequently associated with lipid accumulation in the cytoplasm mainly due to in vitro culture conditions. The objective of this study was to evaluate the lipid composition of biopsied and sexed embryos, produced either in vivo or in vitro from the same Holstein heifers before and after a slow freezing protocol. Lipid extracts were analysed by liquid chromatography-high resolution mass spectrometry, which enabled the detection of 496 features. Our results highlighted a lipid enrichment of IVP embryos in triglycerides and oxidised glycerophospholipids and a reduced abundance in glycerophospholipids. The slow freezing process affected the lipid profiles of IVP and IVD embryos similarly. Lysophosphatidylcholine content was reduced when embryos were frozen/thawed. In conclusion, the embryonic lipid profile is impacted by IVP and slow freezing protocols but not by sex. Lysophosphatidylcholine seemed highly sensitive to cryopreservation and might contribute to explain the lower quality of frozen embryos. Further studies are required to improve embryo freezability by modulating the lipidome.

## Introduction

Embryo biotechnologies are widely used by cattle breeding companies to increase genetic progress by reducing the generation interval and increasing selection intensity. In theory, the most efficient approach would consist in producing numerous in vitro embryos from the best reproducers, biopsy and freeze them to perform their genetic evaluation and transfer into recipients only the most interesting genotypes^1^. However, the low pregnancy rates reported following the transfer of biopsied and frozen in vitro produced embryos, averaging 20% after 60 days of gestation (50% when they were freshly transferred), led to excessive extra-costs and losses of elite embryos, limiting the widespread use of this approach and the achievable annual genetic progress^2,3^.

These low pregnancy rates are partly attributable to the lower quality of in vitro produced embryos compared to those produced in vivo^4–6^. They exhibit a more fragile zona pellucida, more chromosomal abnormalities and a modulated metabolism^7,8^. Differences between the two embryonic origins are partly based on metabolic changes affecting their lipid composition and their cryotolerance. Indeed, in vivo embryos exhibited low metabolic activity leading to an absence of lactate production whereas in vitro matured oocytes produced a measurable amount of lactate^9^, likely due to a stress response to suboptimal culture conditions^7^. The addition of foetal calf serum (FCS) to oocyte and embryo culture media stimulates embryonic development and leads to higher blastocyst and hatching rates than a serum-free medium. However, FCS remains an unsanitary/unsafe product of variable composition between production batches. It includes undefined compounds that could be responsible for variability in the oocyte and embryonic media composition, potentially leading to unreproducible results^10^. Moreover, the addition of FCS for embryo development has been associated with an abnormal accumulation of lipid droplets^11,12^ which are strongly correlated with apoptosis in fresh blastocysts^12^ and embryo cryo-susceptibility^11–13^. The cause of this accumulation of lipids in in vitro embryos produced is still unclear.

The high sensitivity to the cryopreservation process also results in low conception rates compared to those obtained from in vivo produced embryos^12^. During cryopreservation, the lipid phase transition, followed by separation, is one of the major causes of cryo-damages in lipid-rich oocytes and embryos^14,15^. Cryopreservation can damage membrane integrity by causing membrane chilling injuries^15,16^. Altogether these effects reduced blastocyst re-expansion and total cell number and enhanced apoptosis rates which led to a decrease in survival rate after freezing^17–19^. Attempts to optimise embryo cryopreservation were mainly based either on the improvement of the cryopreservation techniques to make them less damaging or by modulating embryo composition, especially in lipids, to make them more resistant to the freezing process. Lipids, particularly phospholipids, are major components of mammalian cell membranes and affect the strengthening of membranes for cryopreservation. Cryo resistance of the cells may therefore be improved by making their membrane more fluid^20^. Membrane fluidity can be influenced by fatty acid composition, especially by the level of unsaturation in glycerophospholipids and by the amount of cholesterol present in the membrane.

We, therefore, hypothesised that the difference in quality and/or cryotolerance between in vitro and in vivo produced embryos could be related to a difference in their lipid composition. Thus, the objective of this study was to evaluate, using mass spectrometry, the lipid content of single biopsied bovine grade 1 blastocysts from in vivo and in vitro origin and determine whether the slow freezing process has an impact on their lipid profiles. Moreover, sexing all embryos using biopsies allowed us to evaluate whether lipid profiles can vary according to sex.

## Results

### Embryo production data

An average of five embryo production sessions by OPU-IVF (ovum pick up, followed by in vitro fertilisation) and three embryo collection sessions by flushing the uterine horn were performed to produce embryos needed for the study. These embryos were produced from eight Holstein Heifers in a Latin square design. The OPU-IVF and embryo collection procedures allowed the generation of 95 and 78 grade one (Q1) expanded blastocysts, respectively (Table 1A,B.). The average number of Q1 blastocysts produced in vitro was 2.1 ± 0.3 per donor during OPU-IVF session, compared to 3.4 ± 1.0 per donor during in vivo embryo session.

**Table 1:** A Embryo production data per session of OPU-IVF, (**B**) Embryo production data per session of embryo collection.

(A) OPU-IVF	Mean ± SEM per session	Total	(B) Embryo collection	Mean ± SEM per donor	Total
Nb punctured follicles	16.0 ± 1.2	737	Nb of follicles at first IA	17.1 ± 1.0	360
Nb recovered COC	8.3 ± 0.6	381	Nb of follicles at second IA	12.3 ± 1.5	258
Nb oocytes in IVM	7.1 ± 0.6	326	Nb of CL	10.5 ± 0.5	221
Nb cleaved embryos	6.3 ± 0.5	290	Nb of recovered embryos	5.8 ± 1.5	133
Nb D6 morulas	1.8 ± 0.3	82	Nb of degenerate embryos	1.1 ± 0.4	107
Nb D6 blastocysts	0.2 ± 0.1	94	Nb of morulas	0.5 ± 0.3	12
Nb D7 blastocysts	3.7 ± 0.4	170	Nb of blastocysts	4.1 ± 1.4	95
Nb D7 blastocysts Q1	2.1 ± 0.3	95	Nb of blastocysts Q1	3.4 ± 1.0	78
Nb D7 blastocysts Q2	1.0 ± 0.1	44	Nb of blastocysts Q2	0.7 ± 0.5	20
Cleaved embryos (%)	87.3 ± 3		Viable embryos (%)	80.5 ± 5.1	
D6 M (%)	21.5 ± 3		Degenerate embryos (%)	19.5 ± 5.1	
D6 BL (%)	30.6 ± 4.2		Morulas (%)	9.0 ± 8.0	
D7 BL (%)	51.5 ± 3.8		Blastocysts (%)	71.4 ± 8.1	
D7 BL Q1 (%)	28.8 ± 3.7		Blastocysts Q1 (%)	58.6 ± 8.1	
D7 BL Q2 (%)	22.7 ± 5.1		Blastocysts Q2 (%)	12.0 ± 3.1	

Nb: number; Nb of CL: number of corpus luteum at embryo collection procedure; M: morula; BL: blastocyst*;* D6 and D7: day 6 and 7 of development. Q1 and Q2: embryo quality based on International Embryo Technology Society recommendations.

### Lipid composition of bovine embryos

**Figure 1:**
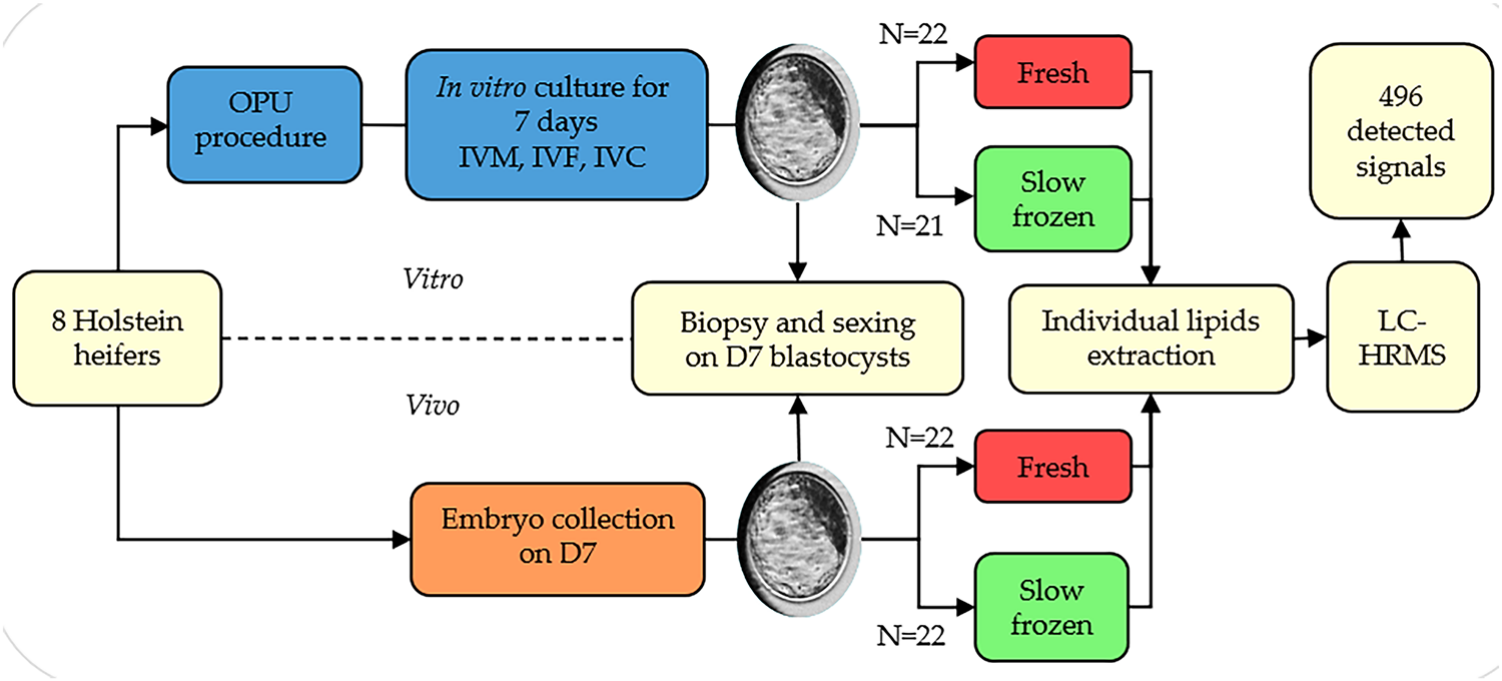
Experimental design. Eight Holstein heifers enabled the production of in vitro and in vivo embryos by OPU-IVF and embryo collection procedures. After seven days of development, all grade 1 expanded blastocysts were biopsied and sexed. Half of each group underwent lipid extraction in a fresh state, the other half were first frozen before undergoing lipid extraction. Lipid extracts were analysed by liquid chromatography-high resolution mass spectrometry and allowed the detection of 496 features.

Liquid chromatography high-resolution mass spectrometry (LC-HRMS) spectra were obtained for 87 individual Q1 expanded blastocysts using ultra-high pressure liquid chromatography) (UHPLC) and 1686 features were identified. After visual inspection of the data, elimination of ions with coefficient of variation (CV) superior to 30% in the quality control (QC) and isotopes elimination, 496 features were conserved. Among those 496 features, 74 were annotated using the SimLipid Database. Annotated lipids are mostly triglycerides (32/74), diglycerides (12/74), glycerophospholipids and lysophospholipids (12/74) (Supplementary Table S1). Among these 87 individual Q1 blastocysts, 43 were produced by OPU-IVF and 44 by embryo collection. Concerning the 43 in vitro embryos, 22 underwent lipid extraction in a fresh state (13 male and 9 female embryos), while 21 were first frozen before undergoing lipid extraction (8 male and 13 female embryos). For embryo collection, 22 underwent lipid extraction in a fresh state (11 male and 11 female embryos), while 22 were first frozen before undergoing lipid extraction (10 male and 12 female embryos) (Fig. 1.).

#### Mass spectrometry lipid signature in fresh embryos produced in vivo or in vitro

**Figure 2:**
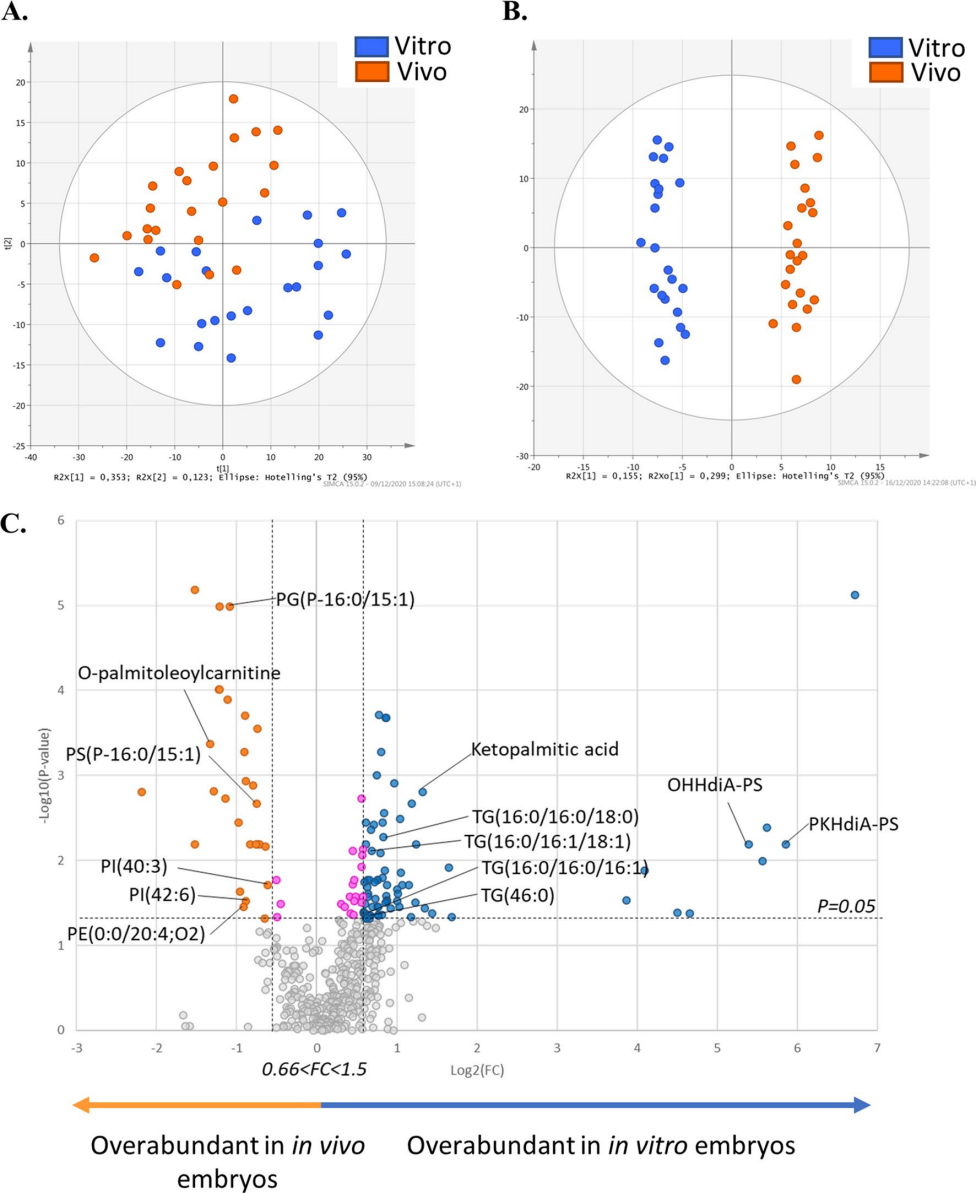
(**A**) Principal component analysis plot (PCA) representing variance among in vivo and in vitro embryos according to principal component analysis. The orange dots show data for embryos with in vivo origin, and the blue dots show data for embryos with in vitro origin. (**B**) Multivariate analysis by orthogonal partial least square discriminant analysis (O-PLS-DA), discriminating the embryonic origins (*vivo* vs. *vitro*) according to the lipid profile of the embryos. (**C**) Univariate analysis by volcano plot based on fold-change and *p* value, highlight several lipids. Blue and orange dots correspond to significantly different lipids between in vitro and in vivo produced embryos. The grey dots represent the non-significant ones. Pink dots correspond to lipids with a significant *p* value but a fold-change between 0.66 and 1.5. All the lipids to the right of this area are over abundant in the in vitro produced embryos (blue dots), while those that are to the left of this area are over abundant in the in vivo produced embryos (orange dots). Statistical significance is determined at *p* < 0.05 and fold-change greater than 1.5 or less than 0.66. Significantly different annotated lipids are represented on the volcano plot by the following abbreviations, phosphatidyl-ethanolamine (PE), inositol (PI), serine (PS), glycerol (PG), triglycerides (TG) and oxidised glycerophospholipid (OHHdia-PS and PKHdiA-PS).

To evaluate the impact of the embryo origin on their lipid profiles, 44 individual fresh blastocysts were used, including 22 in vitro embryos and 22 in vivo embryos (Fig. 1). The principal component analysis (PCA) showed a separation between in vivo (orange dots) and in vitro (blue dots) embryos (Fig. 2A). The orthogonal partial least square analysis (O-PLS-DA) showed a clear discrimination of the two distinct profiles between in vivo (orange dots) and in vitro (blue dots) produced embryos, with a cross-validated predictive ability (Q2) of 0.88 and with good reliability of this model evaluated by CV-Anova, *p* value < 0.0001 (Fig. 2B). The fitted model included 314 features, meaning that most of the identified features (314/496) participated in explaining the lipid profile difference between in vivo and in vitro produced embryos (Supplementary Table S2). The univariate analysis highlighted 105 significantly different features between in vivo and in vitro counterparts (*p* < 0.05 and fold-change < 0.66 or > 1.5), including 50 annotated lipids (Supplementary Table S1). Among the 105 significant features, 27 have a fold-change greater than 2, indicating an increased abundance in in vitro produced embryos, and 12 features exhibited a fold-change lower than 0.5, indicating a decreased abundance in in vitro produced embryos (Table 2). Among these 105 significant features 10 were not found in the fitted model, including 5 annotated lipids, 2 fatty amide, pipericin and N-oleoyl GABA, 1 oxidized glycerophospholipid, OHOHA-PC, and 2 triacylglycerol’s, TG(16:0/16:1/16:1) and TG(46:0). The volcano plot, based on both fold-change and *p* value, highlighted a general lipid enrichment of in vitro produced embryos, particularly in triglycerides (TG) and oxidised glycerophospholipids (OHHdia-PS) which exhibited a fold-change greater than 40 (Fig. 2C). On the contrary, in vivo produced embryos were enriched in glycerophospholipids and particularly in phosphatidyl-ethanolamine (PE), serine (PS), glycerol (PG) and inositol (PI).

**Table 2:** Differentially expressed lipids between in vitro and in vivo produced embryos.

Observed *m/z*	Lipid annotation	FDR p.ajusted	Mean *vitro*	Mean *vivo*	FC *vitro/vivo*	Observed *m/z*	Lipid annotation	FDR p.ajusted	Mean *vitro*	Mean *vivo*	FC *vitro/vivo*
532.3554		1.55E−03	1.79E−03	8.15E−03	0.22	675.2628		4.48E−02	1.80E−04	7.95E−05	2.27
432.2384		6.44E−03	1.23E−02	3.55E−02	0.35	424.2831		3.03E−02	2.78E−04	1.22E−04	2.28
653.2904		6.52E−06	1.96E−05	5.60E−05	0.35	637.3054		3.14E−02	2.08E−04	8.85E−−05	2.35
421.3179	O-palmitoleoylcarnitine	4.26E−04	3.66E−04	9.22E−04	0.40	272.2224		6.44E−03	2.44E−04	1.60E−04	2.50
447.9045		1.71E−02	1.87E−04	4.56E−04	0.41	271.2270	Keto palmitic acid	1.55E−03	4.66E−04	1.86E−04	2.50
1347.8826		1.87E−03	1.45E−04	3.38E−04	0.43	444.3324		3.52E−02	1.23E−04	4.84E−05	2.54
1363.8573		1.87E−03	9.24E−05	2.15E−04	0.43	258.2795		4.22E−02	6.58E−05	2.43E−05	2.71
324.2173		1.02E−05	3.93E−04	9.09E−04	0.43	273.2215		1.02E−02	3.70E−04	1.18E−04	3.14
1342.9274		1.87E−03	1.73E−04	3.82E−04	0.45	444.4207		4.66E−02	1.06E−04	3.28E−05	3.23
382.4050		1.28E−04	1.82E−05	3.92E−05	0.46	326.3786		2.82E−02	8.45E−03	5.78E−04	14.63
708.5122	PG(P-16:0/15:1)	1.02E−05	3.98E−03	8.45E−03	0.47	802.3906		1.22E−02	1.04E−04	6.07E−06	17.09
368.3166		2.07E−02	2.18E−04	1.09E−04	2.00	774.3594		4.04E−02	1.31E−04	5.77E−06	22.66
289.2529		2.96E−02	3.44E−04	1.71E−04	2.01	746.3276		4.22E−02	1.14E−04	4.54E−06	25.20
256.2274		3.52E−02	2.63E−04	1.30E−04	2.02	697.3669	OHHdia-PS	5.32E−03	2.28E−04	5.42E−06	42.08
309.9762		2.78E−03	8.96E−05	4.36E−05	2.05	753.4299		8.26E−03	1.67E−04	3.52E−06	47.34
386.3271		1.31E−02	5.14E−04	2.47E−04	2.08	725.3982		3.77E−03	2.17E−04	4.41E−06	49.20
409.2723		1.94E−02	5.49E−05	2.61E−05	2.10	669.3352	PKHdiA-PS	6.44E−03	1.72E−04	2.98E−06	57.88
654.3331		1.79E−02	2.14E−03	9.63E−04	2.22	532.3839		7.47E−06	6.51E−05	6.17E−07	105.45

In this table only the 39 most different lipids among the 105 different lipids with FC > 1.5 or < 0.66 were presented. *p* values with FDR (false discovery rate) are presented here. The complete table with all the significant features and lipid annotation is provided in Supplementary Table S1.

#### Mass spectrometry lipid signature of in vivo produced embryos before and after slow freezing

**Figure 3:**
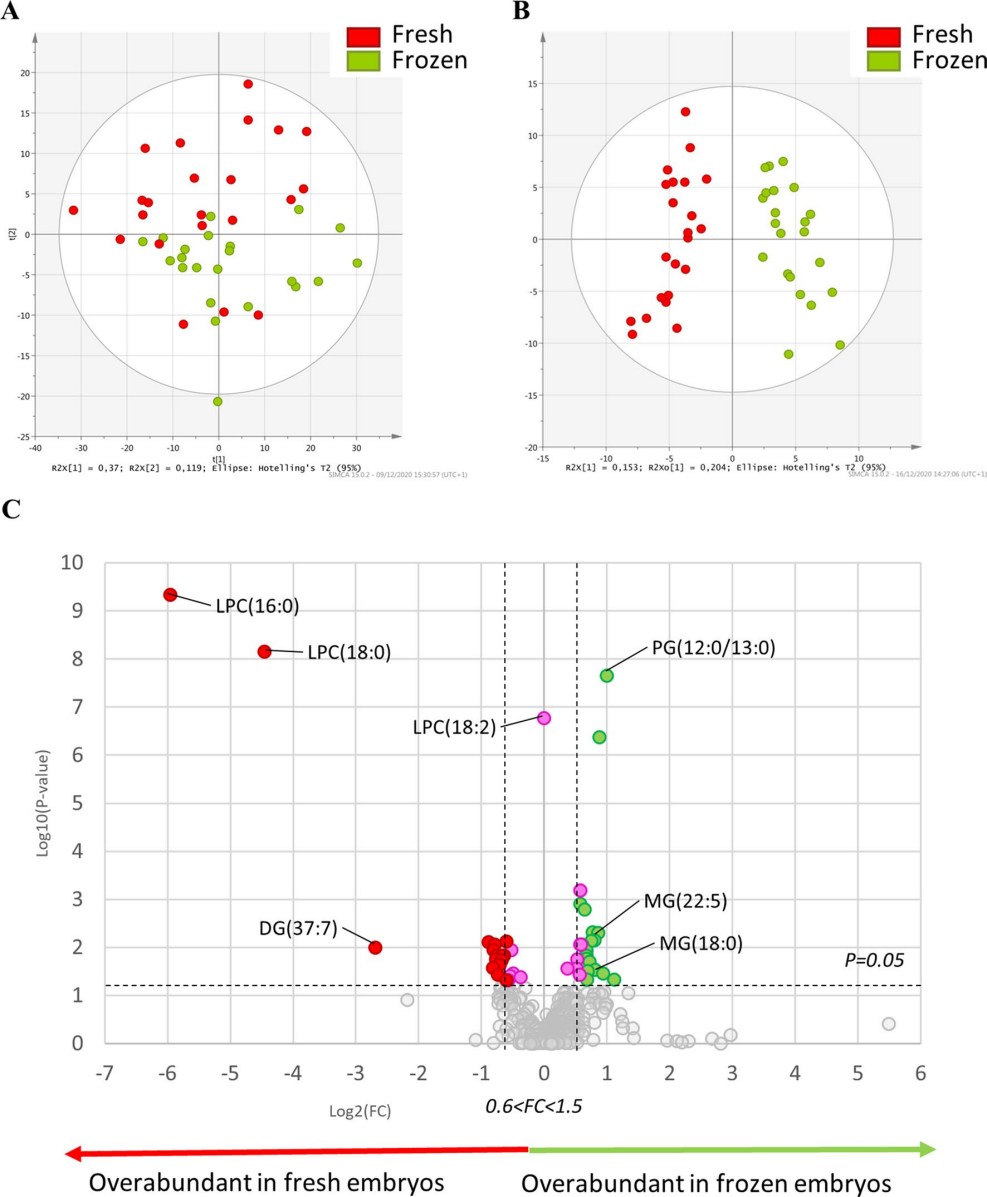
(**A**) Principal component analysis (PCA) plot representing variance among in vivo fresh and frozen embryos according to principal component analysis. The red dots show data for fresh in vivo produced embryos, and the green dots show data for frozen in vivo produced embryos. (**B**) Multivariate analysis by orthogonal partial least square discriminant analysis (O-PLS-DA), discriminating the state before and after freezing (*fresh* vs. *frozen*) according to the lipid profile of the embryos. (**C**) Univariate analysis via volcano plot based on fold-change and *p* value, highlighted several lipids. Red and green dots correspond to significantly different lipids between fresh and frozen in vivo produced embryos. Pink dots correspond to lipids with significant *p* values but a fold-change between 0.66 and 1.5. All the lipids to the right of this area are over abundant in the frozen in vivo produced embryos (green dots), while those on the left of the grey area are overabundant in the fresh in vivo produced embryos (red dots). Statistical significance is determined at *p* < 0.05 and fold-change greater than 1.5 or less than 0.66. Significantly different annotated lipids are represented on the volcano plot by the following abbreviations, lysophosphatidylcholine (LPC), phosphatidylglycerol (PG), monoacylglycerol (MG) and diacylglycerol (DG).

To analyse the effect of the slow freezing process on in vivo produced embryo lipid profiles, 44 individual blastocysts were used, half of them undergoing a slow freezing step, thus generating 22 fresh and 22 frozen in vivo produced embryos. The PCA showed a tendency to distinguish in vivo fresh (red dots) and frozen (green dots) embryos (Fig. 3A). A multivariate analysis (O-PLS-DA) showed a clear discrimination of the two distinct profiles between fresh and frozen in vivo produced embryos, with a cross-validated predictive ability (Q2) of 0.82 and with good reliability of this model evaluated by CV-Anova, *p* value < 0.0001 (Fig. 3B). The fitted model included 162 features that participate in the explanation of the lipid profile difference, between in vivo fresh and frozen embryos (Supplementary Table S2). The univariate analysis highlighted 35 features significantly different between fresh and frozen in vivo produced embryos (*p* < 0.05, FC < 0.66 or > 1.5) (Table 3), including 20 annotated lipids (Supplementary Table S1). Among the 35 significant features, 19 exhibited a fold-change > 1.5, indicating an increased abundance in frozen in vivo produced embryos compared to fresh ones, and 16 features exhibited a fold-change < 0.66, indicating a decreased abundance in frozen in vivo produced embryos compared to fresh ones (Table 3). Among the 35 significant features, 2 were not found in the fitted model, including one eicosanoid, ethyl-diacetoxy-6E,8E,10E,14Z-eicosatetraenoate and one monoglyceride, MG(22:5).

**Table 3:** Differentially expressed lipids between fresh and frozen in vivo produced embryos.

Observed m/z	Lipid annotation	FDR p ajusted	Mean frozen	Mean fresh	FC frozen/fresh	Observed m/z	Lipid annotation	FDR p ajusted	Mean frozen	Mean fresh	FC frozen/fresh
520.3406	LPC(18:2)	1.69E−07	0.00E+00	6.98E−05	0.00	500.2152		7.20E−03	8.80E−05	5.77E−05	1.52
496.3404	LPC(16:0)	4.71E−10	5.25E−06	3.26E−04	0.02	483.2249		1.26E−03	9.88E−05	6.31E−05	1.57
524.3716	LPC(18:0)	7.07E−09	1.21E−05	2.67E−04	0.05	517.2054		1.18E−02	5.99E−05	3.76E−05	1.59
647.4595	DG(37:7)	1.01E−02	5.12E−03	3.31E−02	0.15	443.2687		1.49E−02	3.80E−04	2.38E−04	1.60
310.2381		7.67E−03	4.52E−05	8.33E−05	0.54	528.2464		8.62E−03	1.60E−04	9.97E−05	1.60
349.2158		2.69E−02	2.37E−05	4.18E−05	0.57	424.3641		3.51E−02	1.96E−04	1.22E−04	1.61
653.2904		1.12E−02	3.21E−05	5.60E−05	0.57	369.2409		2.94E−02	5.62E−05	3.48E−05	1.62
547.3459		8.62E−03	1.25E−04	2.16E−04	0.58	518.2176		1.73E−02	5.73E−05	3.47E−05	1.65
357.3368		1.46E−02	2.33E−04	3.93E−04	0.59	427.2913	MG(22:5)	7.04E−03	6.43E−05	3.80E−05	1.69
518.3694		1.77E−02	3.85E−05	6.55E−05	0.59	596.2351		1.62E−03	6.09E−05	3.55E−05	1.71
379.2826		3.64E−02	5.02E−05	8.39E−05	0.60	449.2855		4.93E−03	8.58E−05	4.91E−05	1.75
315.3263		1.88E−02	6.11E−05	9.80E−05	0.62	846.2953		2.00E−02	7.79E−05	4.44E−05	1.75
547.3551		1.49E−02	1.34E−04	2.13E−04	0.63	453.3940		4.88E−03	7.11E−05	3.92E−05	1.81
297.3156		1.46E−02	2.87E−05	4.76E−05	0.64	647.3906	PG(12:0/13:0)	4.25E−07	1.27E−04	6.91E−05	1.84
509.2794		4.78E−02	1.04E−03	1.58E−03	0.66	376.3428	MG(18:0)	3.12E−02	7.43E−04	3.86E−04	1.92
571.2501		7.41E−03	1.24E−04	1.89E−04	0.66	625.4083		2.18E−08	4.11E−05	2.05E−05	2.00
774.2747		4.60E−02	8.46E−05	5.63E−05	1.50	654.3331		4.60E−02	2.10E−03	9.63E−04	2.18

Features with fold-change (FC) > 1.5 or < 0.66 are presented here. *p* value with FDR (false discovery rate) is presented here. The complete table with lipid annotated features is provided in Supplementary Table S1.

The volcano plot highlighted the overabundance of lysophosphatidylcholine (LPC) in fresh in vivo produced embryos and the overabundance of two monoglycerides (MG) and one phosphatidylglycerol (PG) in frozen ones (Fig. 3C). Univariate analysis also showed differences in LPC composition, especially for the LPC (18:2), which was only present in fresh embryos and therefore had a fold-change equal to 0.00 (Table 3).

#### Mass spectrometry lipid signature of in vitro produced embryos, before and after slow freezing

**Figure 4:**
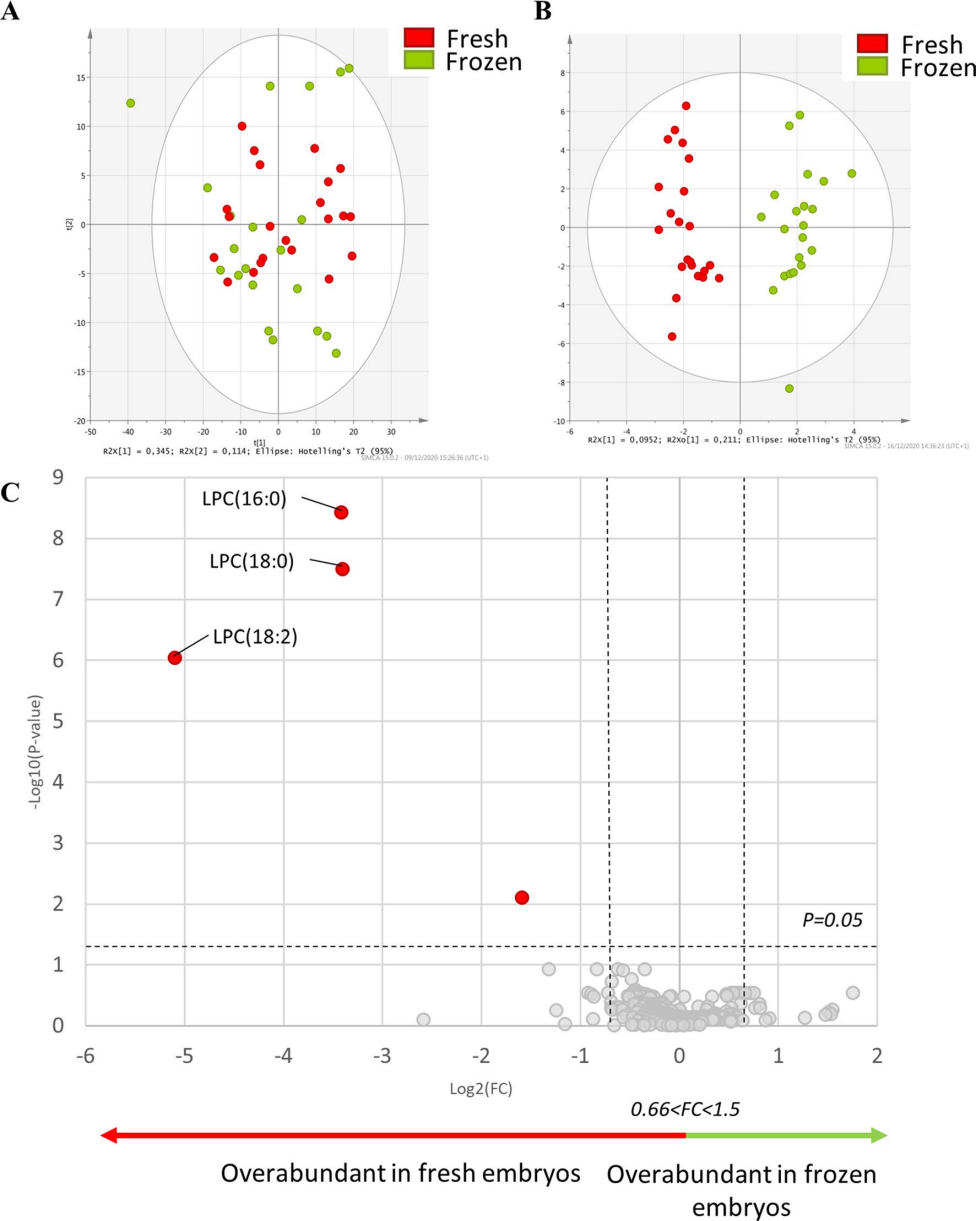
(**A**) Principal component analysis (PCA) plot representing variance among in vitro fresh and frozen embryos according to principal component analysis. The red dots show data for fresh in vitro produced embryos, and the green circles show data for frozen in vitro produced embryos. (**B**) Multivariate analysis by orthogonal partial least square discriminant analysis (O-PLS-DA), discriminating the state before and after freezing (*fresh* vs. *frozen*) according to the lipid profile of the embryos. (**C**) Univariate analysis via volcano plot based on fold-change and *p* value, highlighted several lipids. Red dots correspond to significantly different lipids between fresh and frozen in vitro produced embryos, particularly to those overabundant in fresh embryos, while the grey ones represent the non-significant ones. Statistical significance is determined at *p* < 0.05 and fold-change greater than 1.5 or less than 0.66. Significantly different annotated lysophosphatidylcholine (LPC) are represented on the volcano plot.

To analyse the impact of the slow freezing process on in vitro produced embryo lipid profiles, 40 individual blastocysts were used, half of them undergoing a slow freezing step, thus generating 22 fresh and 21 frozen in vitro produced embryos. The PCA showed overlapping groups between in vitro fresh and frozen embryos (Fig. 4A). Multivariate analysis (O-PLS-DA) showed two distinct profiles, with a cross-validated predictive ability (Q2) of 0.82 and with good reliability of this model evaluated by CV-Anova, *p* value < 0.0001 (Fig. 4B). The fitted model included 47 features that participate in the explanation of the lipid profile difference between in vitro fresh and frozen embryos (Supplementary Table S2). The univariate analysis highlighted four lipids significantly different between fresh and frozen in vitro produced embryos (Table 4), including three annotated as LPC (Supplementary Table S1). All the 4 significant features have a fold-change < 0.66, indicating a decreased abundance in frozen in vitro produced embryos compared to fresh ones (Table 4). The volcano plot highlighted the overabundance of LPC in fresh in vitro produced embryos (Fig. 4C).

**Table 4:** Differentially expressed lipids between fresh and frozen in vitro produced embryos.

Observed m/z	Lipid Annotation	FDR p.ajusted	Mean frozen	Mean fresh	FC frozen/fresh
273.221527		7.99E−03	1.23E−04	3.70E−04	0.33
520.340576	LPC(18:2)	9.02E−07	3.13E−06	1.07E−04	0.03
496.340363	LPC(16:0)	3.77E−09	5.04E−05	5.39E−04	0.09
524.371613	LPC(18:0)	3.16E−08	4.02E−05	4.25E−04	0.09

Features with fold-change (FC) > 1.5 or < 0.66 are presented here. *p* value with FDR (false discovery rate) is presented here. The complete table with lipid annotations is provided in Supplementary Table S1.

#### Mass spectrometry lipid signature in male and female embryos

**Figure 5:**
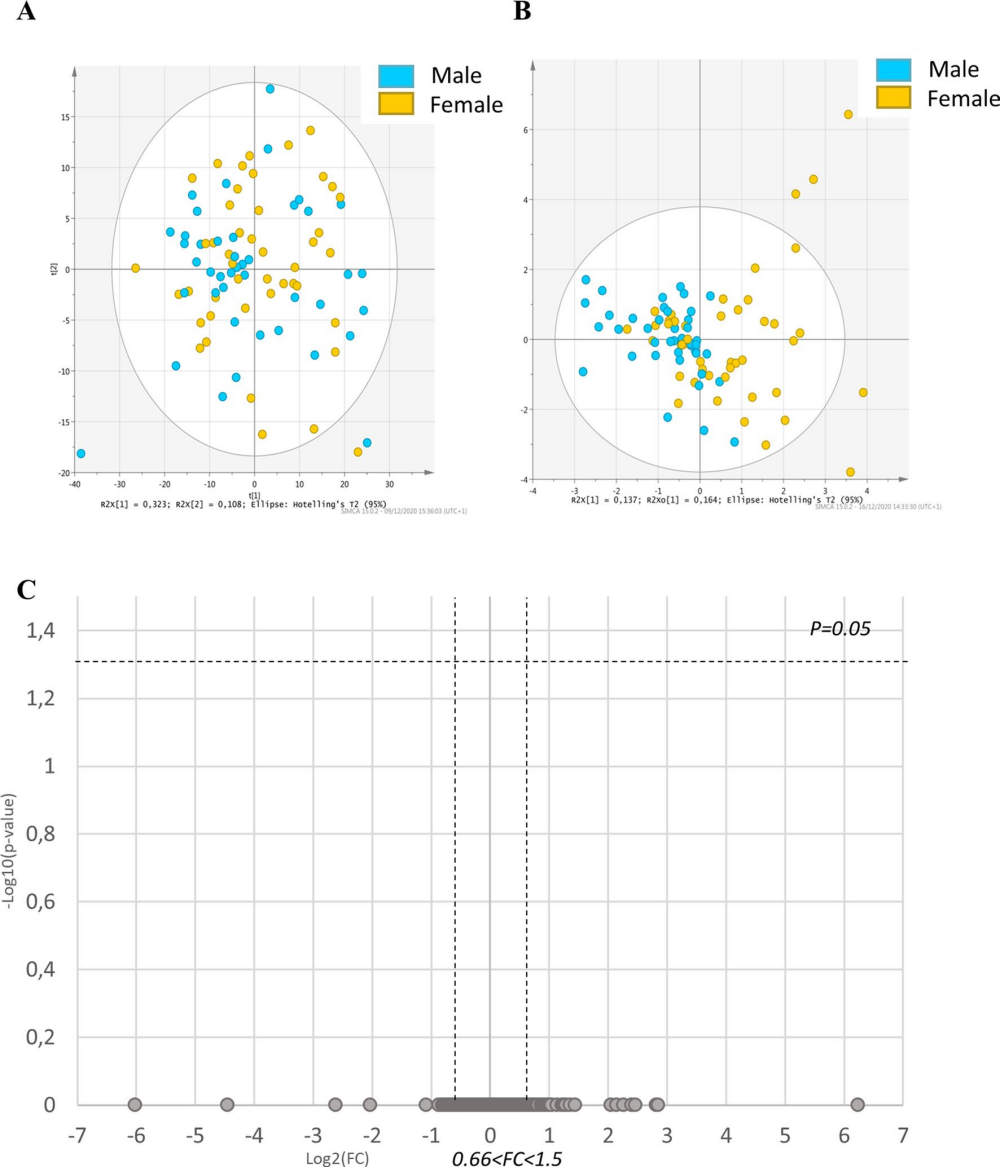
(**A**) Principal component analysis (PCA) plot representing variance among male and female embryos according to principal component analysis (**B**) Multivariate analysis by orthogonal partial least square discriminant analysis (O-PLS-DA), discriminating male and female embryos according to their lipid profile. The light-blue dots show data for male embryos, and the yellow dots show data for female embryos. (**C**) Univariate analysis via volcano plot based on fold-change and *p* value, did not highlight any lipids. The grey dots represent the non-significant lipids. Statistical significance is determined at *p* < 0.05 and fold-change greater than 1.5 or less than 0.66.

To analyse the impact of sex on embryo lipid profiles, all blastocysts were sexed (n = 80). The PCA showed overlapping groups between male and female embryos (Fig. 5A). Sex does not impact lipid profile, in fact, multivariate analysis (O-PLS-DA) showed overlapping groups with a low cross-validated predictive ability (Q2) of 0.20 and CV-Anova, *p* value = 0.0009 (Fig. 5B). The univariate analysis did not show any difference between male and female embryo lipid profiles (Fig. 5C).

## Discussion

This present work is the first, to our knowledge, to evaluate the lipid composition of biopsied embryos, produced either in vivo or in vitro from the same Holstein heifers before and after slow freezing protocol. The final objective of this approach will be to have an interventional action to improve the lipid profile of in vitro embryos. This first step described the differences in embryo lipid profile according to the origin, the freezing/thawing process and the sex. Our results suggested that embryo lipid profile is mainly impacted by in vitro culture protocols and then by the slow freezing process but not by the sex of the embryos.

**Figure 6:**
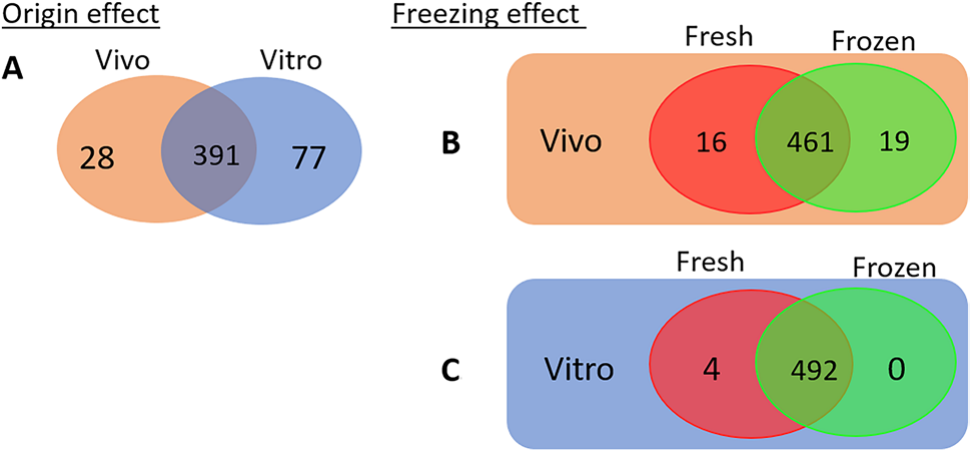
(**A**) Representation of differential lipids between fresh in vivo and in vitro embryos through a Venn diagram. In vivo produced embryos are represented by an orange ellipse and in vitro produced embryos are represented by a blue ellipse. (**B**) Representation of lipid differentials between fresh (red ellipse) and frozen (green ellipse) in vivo produced embryos through a Venn diagram. (**C**) Representation of lipid differentials between fresh (red ellipse) and frozen (green ellipse) in vitro produced embryos through a Venn diagram.

Lipid profile is greatly modulated by the embryo origin, meaning that in vitro culture conditions involve strong modifications in lipid metabolism in comparison with the physiological maternal environment (Fig. 6A). Multivariate analysis revealed that 314/496 features were included in the fitted model, which means that most of the identified features participated in explaining the lipid profile differences between in vivo and in vitro produced embryo and not only the significantly different lipids. Indeed, in vitro produced embryos are enriched in lipids compared to in vivo produced embryos, especially in triglycerides and oxidised glycerophospholipids. The composition of the culture medium can change the amount of fat, in oocytes and embryos, particularly when the serum is added to the medium^11,21^. Such an increase in triglycerides had previously been reported after in vitro culture^22^. Fergusson and Leese demonstrated that an addition of 10% of serum in the culture medium of four-cell stage bovine embryos led to a significant increase in the triglyceride level. The addition of 5% of foetal calf serum (FCS) in culture media also allows the increase in lipid content, like palmitic, palmitoleic, oleic and stearic acid^21^. Even a low amount of 1% serum, used in the present experiment to produce in vitro embryos is sufficient to cause lipid accumulation in embryos. Lipid accumulation can be explained by the absorption of serum lipoproteins^21^, by the neosynthesis of triglycerides due to the presence of serum^23^ or by a reduction in the β-oxidation function in the mitochondria, which compromise the embryo ability to properly metabolize lipids^17,24^. Triglycerides are the major lipid class found in mammalian cytoplasm cells, they are stored as lipid droplets and provide the energy required to support early embryonic development^25^. Moreover, the overabundance of oxidised glycerophospholipids can also be explained by the in vitro culture conditions. In fact, it has been demonstrated that the addition of growth factors, hormones and serum during oocyte maturation stimulated oxidative metabolism and thereby oxidation of fatty acids derived from the breakdown of triglycerides, in the Krebs cycle^26,27^. Such modulations of the lipid profile are therefore likely related to the in vitro culture conditions.

Large amounts of intracellular lipids can compromise embryo development^28,29^ and survival after the cryopreservation process^30^. Therefore, the reduction in these lipid amounts has been the subject of considerable effort^1,2^. Thus, the addition of culture media with the main lipids present in the serum leads to decreased mature oocyte rates and blastocyst rates as well as an increased number of apoptotic cells^31^. In the absence of serum, the first and fourth cell cycles were prolonged by 4–5 h during IVM-IVF, whereas the presence of serum during the culture decreased the duration of the fourth cell-cycle and triggered premature blastulation^4^. One solution may be found in the use of synthetic serum substitute. In fact, phenazine ethosulfate had similar results to those produced by the addition of FCS, while decreasing embryo lipid droplet accumulation when it was added to the embryo culture medium and enhance re-expansion and hatching rate after cryopreservation^12,32–34^.

An alternative explored by several authors is the deprivation of serum over the last 24 h of embryo culture^12^. It is known that serum deprivation represents a stimulus for lipolysis leading to an increase in free fatty acids, and their consumption through β-oxidation for energy production^35,36^. The addition of lipolytic agents is another non-invasive technique allowing the reduction of the level of intracellular lipid content. Among the lipolytic agents, epinephrine, norepinephrine, isoproterenol, forskolin and others had been used to stimulate intracellular lipolysis by directly acting on components of the lipolysis pathway. It has been highlighted that lipolysis induction by addition of 10 µM of forskolin in the culture medium of Bos *indicus* embryos 48 h before vitrification significantly increased the pregnancy rates compared to non-supplemented embryos (18.5% vs. 48.8%)^37^.

In our study, in vivo produced embryos are highly enriched in phospholipids, such as phosphatidylinositol, phosphatidylserine, phosphatidylglycerol and phosphatidylethanolamine. This result is relevant in the literature. Sudano and al. showed that in vivo produced embryos exhibited an overabundance in phospholipids, namely phosphatidylcholine, compared to in vitro ones^38^. As indicated by our results, such an increase in phospholipids is not only restricted to phosphatidylcholine. This overabundance of phospholipids appears to be considered as positive biomarker for successful cryopreservation relative to origin^38^. Phospholipids are the most abundant lipids in the eukaryotic membrane^39^. Particularly, PC, PE and PI are structural units of the membrane, and their concentration determines most of the physicochemical cell membrane properties, like fluidity, permeability and thermal phase behaviour^40^. Therefore, the higher cryosensitivity of in vitro produced embryos could partly be related to their lower phospholipid content.

The causal link between excess of lipids and reduced cryopreservation is still not fully established yet. Although enriched lipid embryos are less resistant to cryopreservation^14^, phospholipids seemed to be important for the cryopreservation process^38^. By enhancing the phospholipid content of in vitro produced embryos through the addition of extracellular vesicles coming from the oviductal fluid, their quality might improve. Banliat and others have shown that extracellular vesicle addition at a concentration of 0.05 mg of proteins/mL in the culture medium induced an overabundance of phospholipids in blastocysts after seven days of culture^41^. Nevertheless, in our study, several lipids were not annotated and it is, therefore, possible that other lipid classes could be affected by the in vitro culture process or by the freezing/thawing of the embryos.

In both in vivo and in vitro produced embryos, fresh embryos are enriched in lysophosphatidylcholine, while only a low amount of those lipids is observed after embryo freezing/thawing. These data suggested that using our slow freezing protocol, LPCs are highly sensitive to cryopreservation. This high sensitivity of LPC is not reported in human plasma, serum and urine where they seemed to remain stable after several freezing (− 20 °C)/warming cycle^42^. LPCs are not only sensitive to the slow freezing protocol but also to vitrification^43^. Indeed, in mice oocytes after vitrification the lysophospholipid content also decreased^43^. Lysophospholipids allow the production of lysophosphatidic acid by enzymes like phospholipase A1, A2 and autotaxin in the bovine embryos and/or from the bovine endometrial and ovarian cells^44,45^. In bovine blastocysts, lysophosphatidic acid stimulates the expression of embryo quality markers, such as insulin-like growth factor 2 receptor (IGF2R) and placenta associated 8 (PLAC8) as well as pluripotency factors like sex-determining region Y box 2 (SOX2) and octamer-binding transcription factor 4 (OCT4), indicating that it can affect bovine embryo quality and support the pluripotency pathway^46,47^. Therefore, such a decrease in the LPC content could contribute to explaining the decrease in survival rates, total cell numbers and pregnancy rates of frozen in vitro produced embryos after transfer. Interestingly, this abundance of LPC in fresh in vivo vs. in vitro produced embryo comparison, was not highlighted by univariate analysis. Nevertheless, in vitro produced embryos have 1.6 time more LPC than in vivo ones. One can speculate that this would be due to in vitro culture media as serum contains LPA as previously described^48^. LPA are small molecules that regulate the Hippo pathway via the inactivation of Lats1/2 kinases (large tumor suppressor 1/2) and the decrease in the phosphorylation of YAP/TAZ which would induce their nuclear translocation and would promote cell differentiation via the increase expression of CDX2^49–51^. If the observed reduction in LPC is related to a decrease in embryo quality and survival, futures studies could focus on the Hippo pathway modulation to improve it.

The lack of data on blastocyst lipid profiles render it difficult to compare our results with the literature. Nevertheless, the lipid profiling that we established between in vivo and in vitro embryos are relevant with the literature, i.e. regarding distribution of triglycerides, therefore strengthening our other data. In vitro systems seemed to smooth the difference between fresh and frozen embryos. This finding strengthened the hypothesis that the reduction in quality between in vitro and in vivo embryos relies upon their lipid profile. Considering that in vitro embryos already exhibited an impaired lipid profile compared to in vivo embryos, the freezing and thawing steps seemed to only mildly affect their lipidome. Indeed, while 35 lipids differed between fresh and frozen in vivo produced embryos, only four lipids differed between fresh and frozen in vitro produced embryos (Fig. 6B,C.). Nevertheless, the great reduction in lysophosphatidylcholine evidenced in both cases might indicate the importance of these lipids for the ability to recover from the cryopreservation process. Their specific cryoprotection might be of interest and the cryoprotectant PEG8000 might be a good candidate to assess in future studies as it was able to prevent LPC degradation in murine oocytes after vitrification^43^.

Metabolic behaviour between male and female bovine embryos differs during the in vitro development. For example, amino acid consumption differs between male and female embryos^52^. In fact, valine seems to be more consumed by female than male embryos. This difference would be due to the activation of the X chromosome. In fact, before its activation, no difference in metabolism has been demonstrated between sexes. In our study, the sex of embryos did not have any impact on the lipid profiles of bovine embryos. However, maternal diet, particularly its lipid content, can influence the sex ratio of offspring born to mice where a very high concentration of saturated lipid allowed a deviation of the sex ratio of pups born in favour of males (0.67)^53^.

This analysis made it possible to compare the entirety of the lipid profiles of each group, but one of the limitations of this technique is that many of the differentially identified lipids are not annotated and are therefore unknown at the present. The discussion of the results was focused on the lipids that could be annotated. Another limitation of this study is that the lipid profiles of embryos was established on all embryos without an assessment of their viability, such assessment would have led to a delay in lipid extraction and therefore to potential modifications of the lipid profiles. It is therefore possible that the lipid profiles presented in the present paper is affected by the viability level of embryos that could be lower after the freezing and/or biopsy steps Nevertheless, the cryosurvival rates and re-expansion rates of additional embryos, contemporary to the present paper, are presented in Supplementary Table S3. Even after freezing and/or biopsy steps, the embryo survival and re-expansion rates are greater than 90%. The lipid profiles presented here can therefore be only mildly affected by a difference in embryo viability. The reduced number of donors is also a limitation. Indeed, the interindividual variation can be high between donors. Nevertheless, to limit this bias a Latin square design was used allowing the donors to be similarly represented in each group. Moreover, performing the analysis on heifers makes it possible to overcome the metabolic and reproductive disturbances linked to milk production interfering with reproductive performance^54^.

The originality of our study enables to prioritize the factors affecting the embryo lipid profile. In this way, future studies should focus on culture conditions and freezing protocols so that they exhibit a lower impact on the lipid profiles of the embryos. The novelty also lies in the fact that we analysed the embryos individually, which enabled to have a more precise embryonic lipid profile and to highlight the interindividual variability. For example, a freezing media preventing the degradation of lysophospholipids could contribute to improving the freezing protocol^47^. Further studies are necessary to investigate the relationship between lipids and both embryo quality and sensitivity to cryopreservation.

## Methods

All chemicals were purchased from Sigma-Aldrich unless otherwise stated.

### Experimental design

All experimental protocols were conducted following the European Directive 2010/63/EU on the protection of animals used for scientific purposes and approved by the French Ministry of National Education, Higher Education, Research and Innovation after ethical assessment by the local ethics committee “Comité d’Ethique en Expérimentation Animale Val de Loire” (protocol registered under APAFIS number 20013-2019032818243107v2). The present study is also in accordance with the ARRIVE guidelines.

To produce in vitro and in vivo embryos, eight Holstein heifers were subjected both to oocytes collection by ovum pick-up followed by in vitro fertilisation procedures (OPU-IVF) and to embryo collection by non-invasive flushing of uterine horns. Heifers were between 24 and 30 months old and weighed between 480 and 590 kg at the time of collection. To avoid any seasonal or individual effects, the experimental design was performed in Latin square using two groups of four individuals. Between the last OPU and the first embryo collection a 7-week rest period was allowed (and vice versa).

All heifers underwent an average of 3 sessions of embryo collection and five OPU sessions to obtain a minimum of forty expanded grade-1 blastocysts of both origin (44 vivo vs. 43 vitro). A single ejaculate from one bull (FR1532181070, Evolution cooperative, France) of proven fertility was used for all the experiments (in vivo and in vitro embryo production). All these embryos were biopsied and half of them were frozen (n = 20) for further lipid extraction while the remaining half was used for immediate lipid extraction in a fresh state (n = 20). Thus, four experimental groups of biopsied embryos were constituted: in vivo frozen embryos, in vivo fresh embryos, in vitro frozen embryos and in vitro fresh embryos (Fig. 1).

### Oestrus synchronization

To produce embryos, experimental heifers were subjected to oestrus synchronisation and ovarian stimulation protocols before either artificial insemination (in vivo embryos) or OPU sessions (in vitro embryos). Synchronisation treatments were performed by insertion of an intravaginal progesterone releasing device (Prid Delta, 1.55 g, Ceva, Libourne, France) and followed, six days later, by 2 mL intramuscular injection of a prostaglandin F2α analogue (Estrumate, MSD Santé Animale—Intervet, France; equivalent to 0.5 mg cloprostenol). The removal of the intravaginal device was performed 24 h after cloprostenol injection. Reference heat was detected by the monitoring of activity and rumination (Heatime, Evolution XY, France) of heifers an average of 48 h after intravaginal device removal. Dominant follicles (follicles with a diameter > 8 mm) were ablated between 8 and 12 days later.

### In vivo embryo production

Ovarian stimulation treatment started 48 h after the removal of the dominant follicles. A new intravaginal progesterone device (Prid Delta, 1.55 g, Ceva, Libourne, France) was inserted and the ovarian stimulation was performed by 8 intramuscular injections of decreasing pFSH/pLH doses (Stimulfol, Reprobiol, Ouffet, Belgium), every 12 h, over four days.

- Day 1: 7 a.m and 7 p.m–60 µg FSH/12 µg LH
- Day 2: 7 a.m and 7 p.m–50 µg FSH/10 µg LH
- Day 3: 7 a.m and 7 p.m–40 µg FSH/8 µg LH
- Day 4: 7 a.m and 7 p.m–25 µg FSH/5 µg LH

Luteolysis was induced with 2 mL intramuscular injection of a prostaglandin F2α analogue (Estrumate, MSD Santé Animale—Intervet, France; equivalent to 0.5 mg cloprostenol), together with the fifth pFSH injection. The PRID DELTA device was removed just after the sixth pFSH injection. All females were artificially inseminated twice with the same frozen-thawed semen 12 and 24 h after oestrus detection by the monitoring of activity and rumination (Heatime, Evolution XY, France). The semen used for all insemination came from the same ejaculate of one bull of proven fertility (GIAGI, FR1532181070, Evolution cooperative, France). Seven days after the first AI embryo collections were performed. For all embryo collections, an epidural injection of 3–5 mL (1 mL/100 kg) of Procamidor (procaine; Richter Pharma, Austria) was performed and the anogenital area was carefully cleaned with an iodine povidone diluted solution (Vétédine solution, Vétoquinol, France). Before collection, heifers were examined by rectal palpation or by an ultrasound exam to evaluate the number of corpus lutea. A flushing solution (Euroflush; IMV Technologies, France) was warmed and maintained in a water bath at 35 °C during the duration of the collection. A cervical dilator was introduced for a few minutes into the cervix. Then, the three-way collection catheter was introduced through the cervix into the first horn 10 cm beyond the uterine bifurcation; the cuff was then inflated with 10 to 12 mL of air. The uterine horn was flushed using 500 mL flushing solution and collected back in a single-use embryo filter (Miniflush Minitübe, Germany). Thereafter, the three-way collection catheter was withdrawn back and the same procedure was used for the second horn. After each procedure, a 2 mL intramuscular injection of a prostaglandin F2α analogue (Estrumate, MSD Santé Animale – Intervet, France; equivalent to 0.5 mg cloprostenol) was given to flushed females to avoid any pregnancies and return to oestrus.

After microscopic evaluation, embryos were classified for quality and stage of development according to the International Embryo Technology Society recommendations (Chapter 9 and Annex D, IETS Manual, 3rd edition). Only grade-1 expanded blastocysts were used for the experiment i.e., expanded blastocysts with compact cell mass, uniformly colored blastomeres, few irregularities or excluded cells and an intact and smooth zona pellucida. Embryo collection was performed every 42 days for up to 3 collections per heifer.

### In vitro embryo production

Ovarian stimulation was performed 36 h after the removal of the dominant follicles. A new intravaginal progesterone device (Prid Delta, 1.55 g, Ceva, Libourne, France) was inserted, and the ovarian stimulation was performed by 5 intramuscular injections of decreasing pFSH/pLH doses (Stimulfol, Reprobiol, Ouffet, Belgium), every 12 h, over 2.5-days.

- Day 0.5: 7 p.m–75 µg FSH/15 µg LH
- Day 1: 7 a.m–62.5 µg FSH/12.5 µg LH
- Day 1: 7 p.m–50 µg FSH/10 µg LH
- Day 2: 7 a.m–37.5 µg FSH/7.5 µg LH
- Day 2: 7 p.m–25 µg FSH/5 µg LH

Cumulus oocyte complexes (COCs) were collected by OPU. After performing locoregional anaesthesia by injection of 3 to 5 mL (1 mL/100 kg) (Procamidor, Richter Pharma, Wels, Austria), the anogenital area was cleaned and disinfected with an iodine povidone diluted solution (Vétédine solution, Vétédine Savon, Vétoquinol, Lure, France). A guide containing an ultrasonographic probe was inserted into the vagina and the ovary was placed by the technician transrectally against the probe (probe EC123, 7.5 MHz, echograph MyLab30, ESAOTE Pie Medical, Saint-Germain-en-Laye, France). Before the puncture, a needle holder, including a needle (18G) and linked to a suction system, was rinsed in flushing solution added with heparin (Heparin Choay, 25,000 UI/5 mL, Sanofi-Aventis, France) (1:50) to prevent the formation of blood clot in the tubing. Then, the needle holder was introduced transvaginally^55^. Follicles from both ovaries, from 6 to 12 mm in diameter, were aspirated and recovered in a 50 mL Falcon tube, containing 1 mL of flushing solution added with heparin (1:50), maintained at 37 °C. OPU-IVF was performed every two weeks for up to an average of 5 sessions per heifer.

#### In vitro maturation

The recovered COCs were selected under a stereomicroscope. Only COCs with homogenous, non-granular cytoplasm and having at least 3 layers of granulosa cells were used^56^. The COCs were washed at 37 °C, three times in embryo flushing media (Euroflush, IMV Technologies, France). The fourth time, COCs were washed in in vitro maturation medium, which consisted in TCM-199, supplemented with 10% FCS (v/v), 10 μg/mL pFSH/pLH, 1 μg/mL 17β-œstradiol, 5 ng/mL epidermal growth factor and 5 μg/mL gentamicin. All COCs were incubated at 38.5 °C for 22 h under a maximum humidity atmosphere of 5% CO_2_ in the air.

#### In vitro fertilisation

Motile spermatozoa were obtained by centrifuging thawed semen on a Percoll gradient composed with two solutions (40% vs. 80%) (Bovidilute and Bovipure, Nidacon, Suede) for 20 min at 500 g. In parallel, matured oocytes were washed two times and then fertilised in 500 µL of a modified Tyrode’s bicarbonate buffered solution (Fert TALP) containing 10 µg/mL heparin, 6 g/L BSA, 20 µM penicillamine, 10 µM hypotaurine, 1 µM epinephrine and 20 µM sodium pyruvate^57^. A single ejaculate from one bull (GIAGI, FR1532181070, Evolution cooperative, France) of proven fertility was used for both in vitro and in vivo embryo production. Percoll treated spermatozoa were coincubated with COCs at 10^6^ spermatozoa/mL at 38.5 °C for 18 h in a maximal humidified atmosphere of 5% CO_2_ in the air.

#### In vitro embryo development

Eighteen hours after fertilisation all presumptive zygotes were cleared of cumulus cells and spermatozoa attached to the zona pellucida by delivery pipetting. Zygotes were washed twice in synthetic oviductal fluid (SOF, Minitüb, Gmbh, Germany) supplemented with 1% of oestrus cow serum, 2% MEM 100×, 1% BME 50×, 0.33 g/L Na-Pyruvate and 6 g/L fatty-acid-free BSA at 38.5 °C in a maximal humidified atmosphere of 5% O_2_, 5% CO_2_ and 90% N_2,_ and then were cultured in a micro drop (30 µL) covered with mineral oil (Liquid Paraffin, Origio, Måløv, Denmark). Each micro drop contained between 1 and 14 embryos (7.0 ± 0.6) depending on the number of oocytes recovered by OPU.

Cleavage rates were assessed under stereoscopic microscopy at 20× magnification 48 h post-fertilisation (day 2) and blastocyst development rates and embryo quality were recorded at day 6 and day 7.

### Embryo biopsies

Biopsies were performed manually using a three-axis hanging joystick oil hydraulic micromanipulator (Narishige, Tokyo, Japan), coupled with an inverted microscope (Nikon eclipse TS100, Tokyo, Japan) and stainless homemade microblade. The homemade microblade was made from a razor blade piece (Moria, Antony, France) stick with glue to a stainless support which is finally fixed to a micromanipulator. For biopsy, expanded grade-1 blastocysts were fixed from ICM side by a holding pipette and then the microblade was placed on the edge of the embryo end, at the opposite from ICM, and moved down to separate an average of 10 trophectoderm cells.

Embryos were micro manipulated on a dish containing 200 µL of embryo holding medium (EHM, IMV Technologies, France). The biopsied cells (5–10 blastomeres) used for embryo sexing were removed from the trophectoderm and were transferred to a 0.5 mL vial and immediately cryopreserved on dry ice before being stored at − 80 °C until analysis. To prevent genomic DNA contamination between embryos, the microblade and holding pipette were washed using ethanol–acetic acid and rinsed in a water bath between each biopsy.

After biopsy half of the biopsied embryos from both groups, in vivo (n = 22) and in vitro (n = 22), was randomly transferred in a 1.5 mL vial in a minimum volume of EHM for the lipid extraction experiment, and the other half from both groups, in vivo (n = 22) and in vitro (n = 21) was frozen.

### Embryo freezing

For the slow freezing procedure, embryos were washed in EHM and placed in 1.5 M ethylene glycol embryo freezing medium (ET freezing media, IMV, Technologies, France) with 0.1 M sucrose added for 10 min at room temperature. Embryos were individually mounted in 250 µL straws, respecting the following proportion 4:2:4. The first column was composed of EHM, the second column contained embryos in freezing media, the third column was composed of EHM. The column containing embryos was separated from others by air. Straws were placed in the cryochamber of the freezer (Freeze Control, Cryologic, Australia), previously equilibrated at − 6 °C. After 2 min, the seeding was manually induced. The temperature was stabilised at − 6 °C for 8 min post-seeding and then dropped to -32 °C at a rate of − 0.3 °C/min. At the end of the program, straws were directly plunged in liquid nitrogen and stored for 1 week before thawing and lipid extractions. For thawing, straws were kept 5 s in ambient air and then immersed in a water bath at 35 °C for 30 s. Embryos were washed three times in PBS and transferred in 1.5 mL vials in minimum volumes for lipid extractions.

### Embryo sexing

Sex determination was performed by Y chromosome-specific DNA probe technology coupled with amplification by PCR^58^. Biopsies were lysed in 0.015 mol/L KCl medium supplemented with 2 g/L BSA (Gibco Laboratories, Grand Island, NY). In each tube, 20 µL of buffer containing proteinase K and the two primers were added. All samples were denatured at 95 °C for 15 min to stop the proteinase K action. The reaction medium (20 µL) including the nucleotides and the TAQ polymerase was then added. The mixture was amplified for 29 cycles consisting of denaturation at 95 °C for 30 s, hybridisation at 60 °C for 30 s, and extension at 72 °C for 30 s. After the last cycle, all samples were incubated for a further 5 min to assure complete extension at 72 °C. A total of four controls were processed simultaneously: two male controls (20 and 200 pg male DNA), one female control (100 pg female DNA) and a negative control (no DNA)^59^. PCR products were analysed using an E-gel iBase (Thermo Fisher Scientific, Villebon-sur-Yvette, France).

After migration by electrophoresis, the resulting bands were observed with UV transillumination. The Y-specific primer generated a 148-base pair (bp) fragment in male samples and the internal control primer generated a 443 bp fragment in all samples. No band was revealed in the negative control. After visualisation, the samples generating two bands corresponded to male embryos and samples generating only one band were female embryos.

### Lipid analysis by mass spectrometry, sample preparation and data acquisition

The liposoluble fraction of each embryo, after trophectoderm biopsy, was individually extracted based on a modified Bligh and Dyer method^60^. Briefly, 425 µL of diluted methanol in sterile water (75:25) was added to the embryos. Then, the addition of 400 µL of chloroform and 275 µL of sterile water was performed. The mix was then thoroughly vortexed before centrifugation at 10,000×g for 10 min at 4 °C. The lower phase (350 μL) corresponding to the nonpolar fraction was recovered and put in glass tubes for further solvent evaporation in a SpeedVac (Thermo Fisher Scientific, Waltham, MA) for 45 min at room temperature. The residue was then reconstituted with 100 μL of a 6:3:1 mix of acetonitrile (ACN)/water/isopropanol followed by centrifugation (15,000×*g*, 10 min, 4 °C) before mass spectrometry analysis.

LC-HRMS analysis was performed as described by Beauclercq and al.^61^. Briefly the analysis was performed on a UHPLC Ultimate 3000 system (Dionex, Sunnyvale, CA), coupled to a Q-Exactive mass spectrometer (Thermo Fisher Scientific, Waltham, MA) and operated in positive ionisation mode (ESI+). Chromatography was carried out with a 1.7 μm C18 (150 mm × 2.10 mm, 100 Å) UHPLC column (Kinetex, Phenomenex, Torrance, CA) heated at 55 °C. The solvent system comprised mobile phase A [isopropanol/ACN (9:1) + 0.1% (vol/vol) formic acid + 10 mM ammonium formate], and mobile phase B [ACN/water (6:4) + 0.1% (vol/vol) formic acid + 10 mM ammonium formate]; the gradient operated at a flow rate of 0.26 mL/min over a run time of 24 min. The multistep gradient was programmed as follows: 0–1.5 min-32–45% A, 1.5–5 min-45–52% A, 5–8 min-52–58% A, 8–11 min-58–66% A, 11–14 min-66–70% A, 14–18 min-70–75% A, 18 to 21 min-75–97% A and 21–24 min-97% A. The autosampler temperature (Ultimate WPS-3000 UHPLC system, Dionex) was set at 4 °C, and the injection volume for each sample was 5 µL. The heated ESI source parameters were a spray voltage of 3.5 kV, a capillary temperature of 350 °C, a heater temperature of 250 °C, a sheath gas flow of 35 arbitrary units (AU), an auxiliary gas flow of 10 AU, a spare gas flow of 1 AU and a tube lens voltage of 60 V for C18. During the full-scan acquisition, which ranged from 250 to 1600 m/z, the instrument operated at 70,000 resolution, with an automatic gain control target of 1 × 10^6^ charges and a maximum injection time of 250 ms. The instrumental stability was evaluated by multiple injections (n = 9) of a QC sample obtained from a pool of 10 μL of all samples analysed. This QC sample was injected once at the beginning of the analysis, then after every 10 sample injections and at the end of the run.

### LC-HRMS data processing

Galaxy Workflow4metabolomics was used to process the raw data for features detection and retention time correction. Spectra of QC samples was analysed, and each chromatographic peak that differed from the background noise corresponded to a peak. Features with variance intensities greater than 30% in QC samples or with QC variance superior to sample variance were removed as well as those identified as background noise or poorly integrated after visual inspection^62^. Lipid annotation was performed on SimLipid, a high throughput lipid identification and quantification software. The peak intensities were normalised by the total area of the spectra for each sample, transformed into a logarithm using the automatic transformation tool of SIMCA software (Umetrics, Umeå, Sweden). Lipids were annotated (< 0.01 ppm) according to either the homemade SimLipid (highlighted in yellow, in Supplementary Table S1), database or to the LIPID MAPS Lipid Classification System^63,64^ (not highlighted in Supplementary Table S1). (https://www.lipidmaps.org/resources/tools/bulk_structure_searches.php?database=LMSD).

### Statistical analysis of data on lipid profile

PCAs were performed on the data as exploratory unsupervised analysis representing the distribution of embryos, of the different comparisons, according to their lipid profile. The proportion of explained variance is represented by the sum of the R^2^ of the first two components. A higher R^2^ indicated a more accurate model. The ellipse represents the 95% confidence interval (Hotelling’s T-square). Multivariate analysis was performed with orthogonal partial least square (O-PLS-DA) approaches, which were performed on the data set in the form of supervised analysis to predict groups by maximising the explained variance between groups, using the SIMCA Software^65^. Ion selection was carried out by repeatedly excluding the variables with low regression coefficients and wide confidence intervals derived from jack-knifing combined with low variable importance in the projection (VIP) until maximum improvement of the quality of the models. Only features with VIP > 1 are been presented in Supplementary Table S2. For the comparison of the lipid profiles of the in vivo versus in vitro produced embryos, the features with VIP > 1 are presented in the green column of the Supplementary Table S2. For fresh versus frozen in vivo produced embryos features with VIP > 1 are presented in the blue column, and for in vitro embryos, when VIP > 1, features are represented in salmon-color column. Model quality was evaluated with cross-validation by Q^2^ (goodness of prediction) and CV-Anova (cross-validation-analysis of variance). The CV-Anova is a diagnostic tool for assessing the reliability of O-PLS-DA models; the associated *p* value is indicative of the statistical significance of the fitted model. The O-PLS-DA models were performed to show the relationship between variance in the data of the embryo origin (*vivo* vs. *vitro*), the state before extraction (*fresh* vs. *frozen*) and the sex of the embryos (*male* vs*.* *female*). The differentially expressed lipid species between each comparison were identified using nonparametric Wilcoxon tests with FDR correction and a fold-change greater than 1.5 and less than 0.66, on Metaboanalyst (https://www.metaboanalyst.ca/)*.* All lipids significantly different are presented in Supplementary Table S1. For each comparison, in vivo versus in vitro produced embryo (green column), in vivo fresh versus frozen (blue column) and in vitro fresh versus frozen embryos (salmon-color column), *p* value, average peak intensity and fold-change are presented. Univariate analysis is represented by a volcano plot combining fold-change and *p* value of the *t*-tests. The x-axis represents the fold-change between the subject groups (base 2 logarithm scale) and the y-axis represents the *p* value for the t-test of differences between the variables (negative base 10 logarithm scale).

## Supplementary Information

Supplementary Information 1.

Supplementary Information 2.

Supplementary Information 3.
