# Supplementary material for: Lipid profile of bovine grade-1 blastocysts produced either in vivo or in vitro before and after slow freezing process
Source: Sci Rep. 2021 Jun 2;11:11618. doi: 10.1038/s41598-021-90870-8 (PMC8172931; doi:10.1038/s41598-021-90870-8)
Supplement: Supplementary file 3 — Supplementary Information 3. [file 41598_2021_90870_MOESM3_ESM.pdf]

Supplementary Table 3: Embryonic survival rates (mean ± SD) of *in vitro* produced grade-1 expanded blastocysts

	n embryos	re-expansion rate after 24H	re-expansion rate after 48H	% dead cells (n embryos)
fresh embryos	29	100	100	
frozen embryos	55	84,3 ± 16,4	91,55 ± 12,1	5,2 ± 3,4 (16)
biopsied fresh embryos	21	95,45 ± 6,43	95,45 ± 6,43	
biopsied frozen embryos	93	94,96 ± 6,20	93,34 ± 7,53	

Embryonic survival was assessed on additional embryos, contemporary to the present study. After the same IVM, IVF and IVC steps, day 7 blastocysts were recultivated either freshly or after the same freezing and/or a biopsy steps as described in the present paper. After seven day of culture, embryos were graded and only grade 1 expanded blastocysts were used. Then, the embryos were recultivated for 48h, whole (n=29) or biopsied (n=21). This allowed to highlight the effect of recultivation up to D9 of development with or without biopsy step. Some other embryos, at D7, were frozen either whole (n=55) or after biopsy (n=93). After thawing, the cryosurvival rate of the expanded grade-1 blastocysts was evaluated by re-expansion rate after recultivation over 48h. This allowed to highlight combined effect of slow freezing protocol and recultivation, with or without biopsy step. Re-expansion rates were assessed after 24 and 48H by morphological assessment using. The percentage of dead cells is measured using Live/dead protocol (InVitrogen, Paisley, UK), following the manufacturer recommendations, and cell counting under confocal microscope observation after 48h of embryo recultivation after thawing.
